# Complement inhibitor CSMD1 acts as tumor suppressor in human breast cancer

**DOI:** 10.18632/oncotarget.12729

**Published:** 2016-10-18

**Authors:** Astrid Escudero-Esparza, Michael Bartoschek, Chrysostomi Gialeli, Marcin Okroj, Sioned Owen, Karin Jirström, Akira Orimo, Wen G. Jiang, Kristian Pietras, Anna M. Blom

**Affiliations:** ^1^ Department of Translational Medicine, Lund University, Malmö, Sweden; ^2^ Department of Laboratory Medicine, Lund University, Lund, Sweden; ^3^ Department of Medical Biotechnology, Medical University of Gdańsk, Gdańsk, Poland; ^4^ Cardiff China Medical Research Collaborative, Cardiff University School of Medicine, Cardiff University, Cardiff, UK; ^5^ Department of Clinical Sciences, Lund University, Lund, Sweden; ^6^ Department of Pathology and Oncology, Juntendo University School of Medicine, Tokyo, Japan

**Keywords:** breast cancer, tumor suppressor, CSMD1, complement system, invasion

## Abstract

Human CUB and Sushi multiple domains 1 (CSMD1) is a membrane-bound complement inhibitor suggested to act as a putative tumor suppressor gene, since allelic loss of this region encompassing 8p23 including CSMD1 characterizes various malignancies. Here, we assessed the role of CSMD1 as a tumor suppressor gene in the development of breast cancer *in vitro* and *in vivo*. We found that human breast tumor tissues expressed CSMD1 at lower levels compared to that in normal mammary tissues. The decreased expression of CSMD1 was linked to a shorter overall survival of breast cancer patients. We also revealed that expression of CSMD1 in human breast cancer cells BT-20 and MDA-MB-231 significantly inhibited their malignant phenotypes, including migration, adhesion and invasion. Conversely, stable silencing of CSMD1 expression in T47D cells enhanced cancer cell migratory, adherent and clonogenic abilities. Moreover, expression of CSMD1 in the highly invasive MDA-MB-231 cells diminished their signaling potential as well as their stem cell-like properties as assessed by measurement of aldehyde dehydrogenase activity. In a xenograft model, expression of CSMD1 blocked the ability of cancer cells to metastasize to secondary sites *in vivo*, likely via inhibiting local invasion but not the extravasation into distant tissues. Taken together, these findings demonstrate the role of CSMD1 as a tumor suppressor gene in breast cancer.

## INTRODUCTION

Breast cancer is the most frequent cancer in women and many pathophysiological mechanisms leading to its development remain to be elucidated. Some of these relate to tumor suppressor genes defined as a group of genes that when down-regulated, mutated or absent contribute actively to carcinogenesis. Inactivation of tumor suppressor genes is a fundamental hallmark of cancer [1] and their functions span from inhibition of cell growth to regulation of the cell cycle [2].

Human CUB and Sushi multiple domains 1 (CSMD1) is a large (∼ 390 kDa) membrane-bound complement inhibitor [3]. It is composed of 14 N-terminal CUB domains separated by single complement control protein (CCP) domains and followed by 15 consecutive CCP domains. It has a single membrane-spanning domain at the C-terminus and a small cytoplasmic tail of 56 amino acids with a putative tyrosine phosphorylation site. Therefore, CSMD1 has been proposed to have an active role in cell cycle regulation and controlling apoptosis, for example via the Smad pathway in melanoma cells [4]. CSMD1 is highly expressed in testis, cerebral cortex, cerebellum and brain white matter. A weaker expression was seen in breast, placenta and thyroid gland [3].

The CSMD1 gene occupies over 2 Mb in the short arm of chromosome 8 (8p23) [5]. Allelic loss, mutations and methylations of this particular region have been reported in malignancies such as breast [6, 7], head and neck [6, 8], oral squamous cell carcinoma [9], prostate [10], colorectal [11–13], liver [14], lung [6] and skin [6] cancer. In addition, decreased CSMD1 expression has been linked to poor prognosis in patients [9, 15, 16]. Thus, CSMD1 was implied to act as a putative tumor suppressor gene [5]. However, these studies have only showed varying expression of CSMD1 by using commercial peptide-specific antibodies or genetic screening for deletions that covers regions with several genes. Therefore, the assumption that CSMD1 acts as tumor suppressor gene remains unsubstantiated with a direct experimental approach.

In the present study, by using *in vitro* and *in vivo* approaches, we aimed to determine whether CSMD1 acts as a tumor suppressor protein in breast cancer progression.

## RESULTS

### CSMD1 expression in human breast cancer tissues is decreased and correlates with poor prognosis

**Figure 1:**
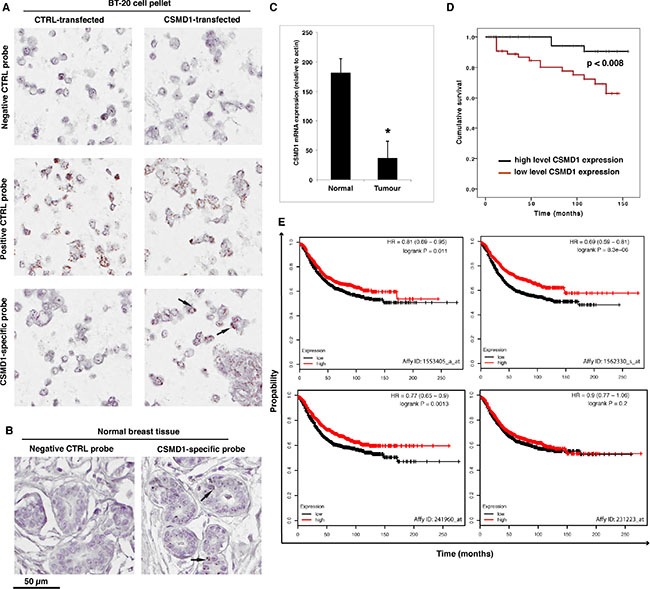
Detection of CSMD1 mRNA in normal breast tissue and quantitation of CSMD1 mRNA transcript in breast cancer tissues CSMD1-specific probe, as well as a negative (DapB) and a positive (PPIB) control probes were included when staining BT-20 expressing CSMD1 and CTRL paraffin-embedded cell pellets for validation of the method (**A**). RNAscope detection of CSMD1 mRNA transcripts in paraffin-embedded normal breast tissue. Samples were hybridised with either CSMD1-specific probe or negative control probe. A positive signal for CSMD1 was observed in the normal breast tissues. The black arrows outlined the mRNA brown dots (**B**). Breast tumor tissues had significantly lower levels of CSMD1 mRNA transcript compared with normal tissues; *p < 0.05 by Mann-Whitney test (**C**). Patients with low levels of the CSMD1 transcript showed a significantly shorter overall survival (log rank test) (**D**). Kaplan–Meier plots using as using recurrence-free survival as an endpoint for four probes of CSMD1; HR, hazard ratio (**E**).

First, we set out to evaluate the expression of CSMD1 in paraffin-embedded normal breast tissue. To validate our approach of using an RNAscope assay for detection of CSMD1 mRNA, we prepared BT-20 cells differing in expression of CSMD1 treated the same way as human tissues. Strong mRNA signal (brown dots) was observed for the positive control (PPIB, cyclophilin) in both BT-20 CSMD1- and control-transfected cells, whereas the CSMD1-specific mRNA signal was only detected in BT-20 expressing CSMD1. No signal was found for the negative control DapB probe (Figure 1A). Further, CSMD1-specific mRNA was detected in normal breast tissue, particularly in ductal epithelial cells (Figure 1B).

Next, we measured the expression of CSMD1 transcript in a cohort of human breast cancer using qPCR. Breast cancer tissues (*n* = 127) had significantly lower levels of the CSMD1 transcript than normal tissues (*n* = 32) (*p* < 0.05) (Figure 1C). Importantly, patients with low CSMD1 levels had a significantly shorter survival compared with those who had high levels (117.5 ± 6.6 month vs 149.3 ± 3.7 months, *p* < 0.008 by log rank analysis) (Figure 1D). Accordingly, tumors with higher Nottingham Prognostic Index (NPI) [17] had statistically significantly lower levels of CSMD1 transcript (133 +/− 14 for NPI < 3.4; 18.6 +/−17.8 for NPI 3.4-5.4; 6.4 +/− 4.9 for NPI > 5.4). These NPI values correspond to 85, 70 and 50% 5-year survival, respectively.

Additionally, analysis of mRNA expression array data for 1600 breast cancer patients with the online survival analysis tool KM plot (kmplot.com) supported the tumor suppressor function of CSMD1 in an independent patient cohort using recurrence-free survival as an endpoint [18]. In this dataset, three out of four probes for CSMD1 showed significant association with recurrence free survival with hazard ratios varying between 0.69 and 0.81 (Figure 1E).

### CSMD1 expression and knockdown in breast cancer cells

**Figure 2:**
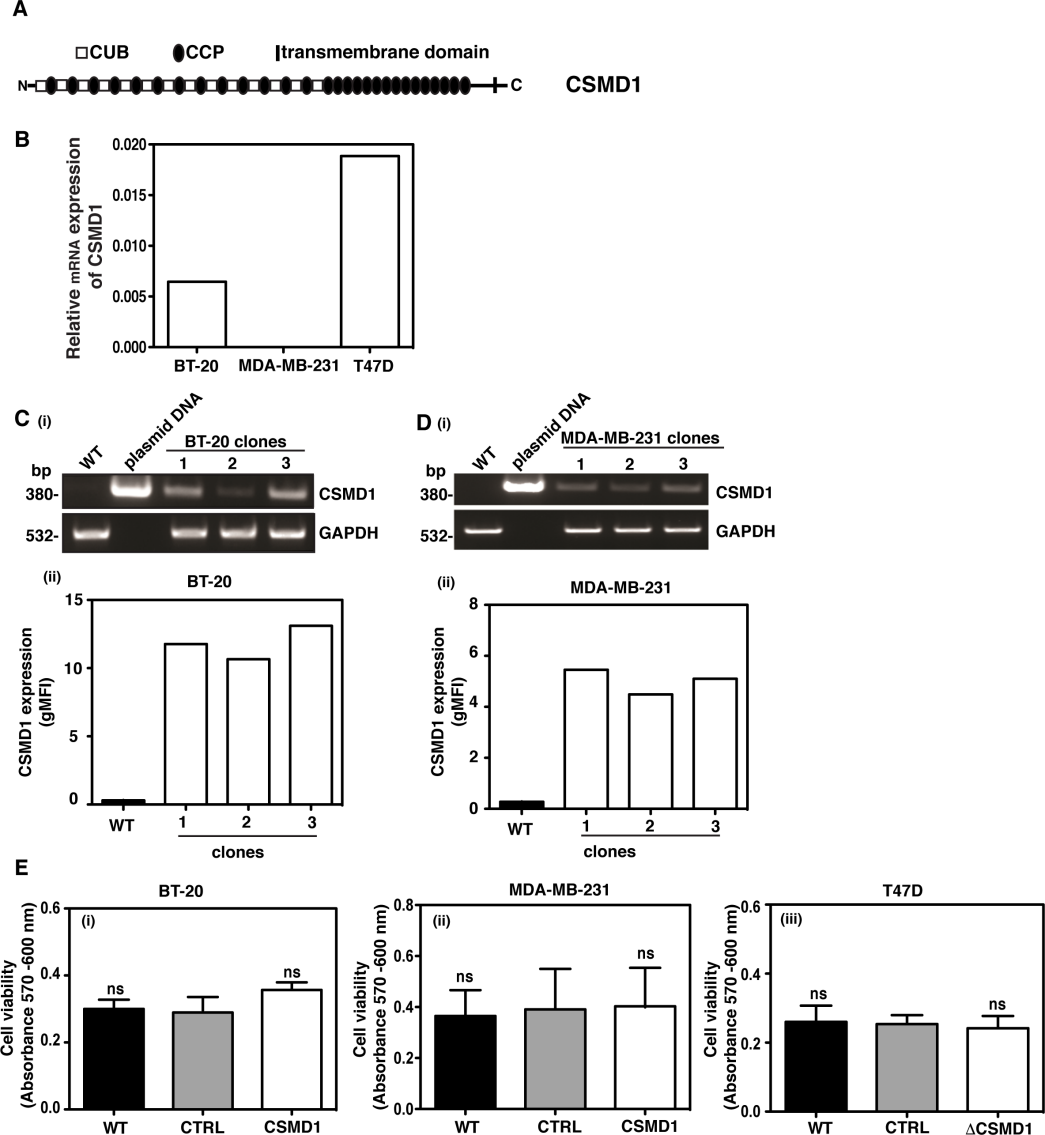
Expression of CSMD1 in breast cancer cell lines CSMD1 is composed of CUB and CCP domains followed by a single transmembrane domain and a small cytoplasmic region (**A**). Screening of breast cancer cell lines for CSMD1 coding sequence at mRNA level using qPCR. The breast cancer cells BT-20 and MDA-MB-231 were selected for expressing CSMD1 and T47D for knocking-down (**B**). Verification of CSMD1 expression in the 1/2/3 clones for BT-20 and 1/2/3 clones for MDA-MB-231 cells by conventional PCR (i) and flow cytometry (ii). CSMD1 levels were higher when compared to the WT. The data presented is representative of a single experiment performed in duplicates (**C**–**D**). The housekeeping gene GAPDH was used as a control of RNA integrity. Cell viability measured after 24h incubation (**E**) was not affected when expressing CSMD1 in the breast cancer cells BT-20 and MDA-MB-231 (i-ii) or when silencing CSMD1 in the breast cancer cell T47D (iii). Values are means ± SD from 3 independent experiments performed in five replicates. One-way ANOVA was used to calculate statistical significance; ns, not significant.

The CSMD1 mRNA expression was examined in three breast cancer cell lines by RT-PCR. Due to low expression levels (Figure 2B), BT-20 and MDA-MB-231 cells were selected for expression of CSMD1. On the other hand, T47D cells expressed appreciable amounts of CSMD1 and were therefore chosen for knocking down CSMD1 expression. Successful expression of CSMD1 in clones 1/2/3 for BT-20 cells (Figure 2Ci) and 1/2/3 for MDA-MB-231 cells (Figure 2Di) was detected by conventional PCR. The expression of CSMD1 was confirmed by flow cytometry analysis with a specific antibody (Figure 2Cii and 2Dii). In order to knock down the expression of CSMD1 in T47D cells, we used a ribozyme transgene generated previously in which a reduction of CSMD1 was confirmed on both the RNA and the protein levels [3].

### Increased CSMD1 expression contributes to the decreased cancer cell migration and invasion

**Figure 3:**
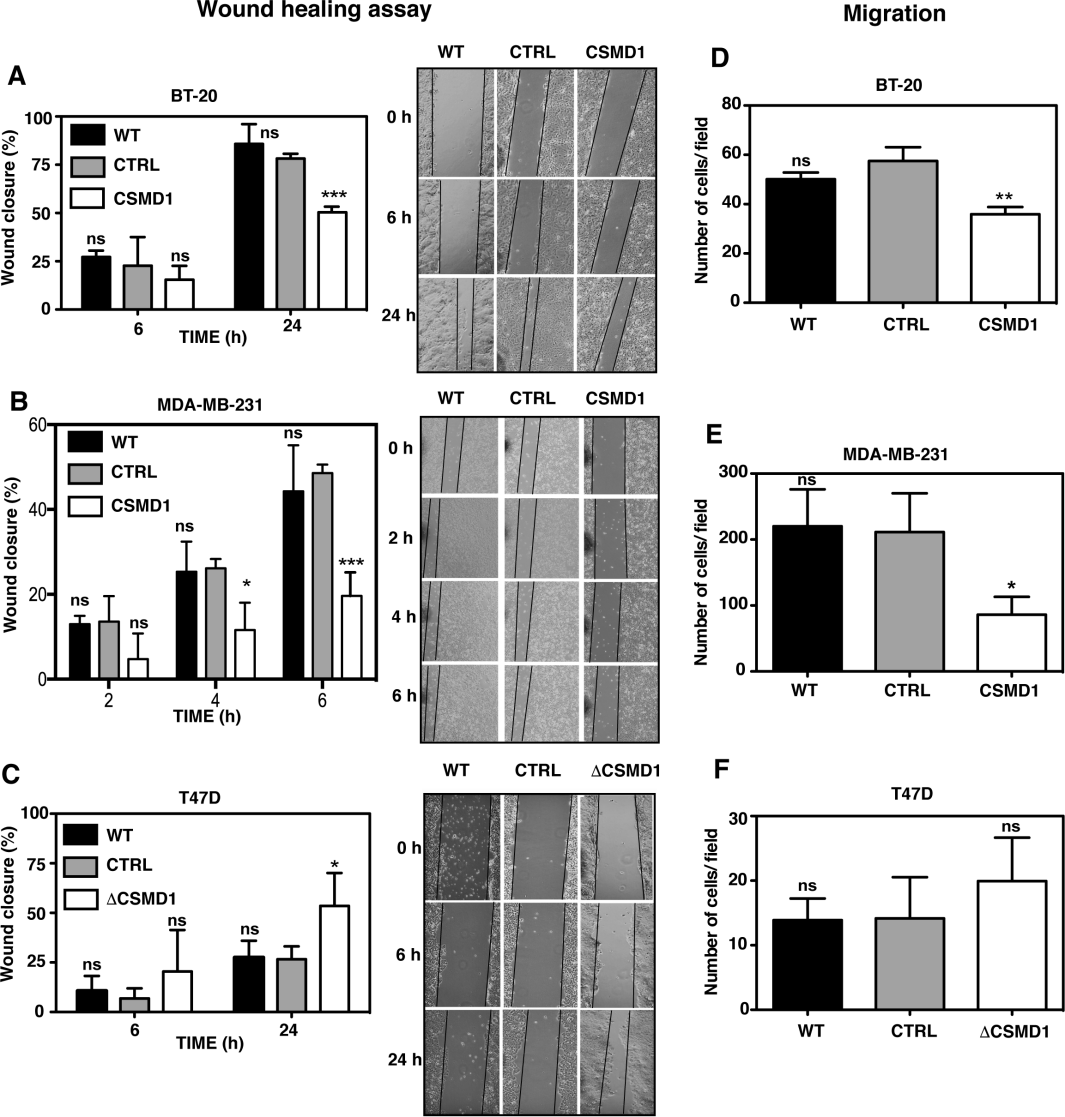
Alteration of CSMD1 expression affects wound healing and migration A monolayer of cells was wounded and photographs were taken at different time points. The wound closure was expressed as percentage of wound closure as compared with the zero time point. Percentage wound closure observed in BT-20 (**A**), MDA-MB-231 (**B**) and T47D cells (**C**). WT, CSMD1 or ΔCSMD1 cells were compared to CTRL cells by two-way ANOVA to calculate statistical significance. Motile cells that passed through the pores and adhered to the underside of the cell culture insert membrane following a FBS gradient were photographed and counted for BT-20 (**D**), MDA-MB-231 (**E**) and T47D cells (**F**). A one-way ANOVA was used to calculate statistical significance between the CTRL cells and WT, CSMD1 or ΔCSMD1 cells; *p < 0.05; **p < 0.01; ***p < 0.001; ns, not significant. Results shown are mean of cells counts ± SD from 3 independent experiments performed in duplicates.

No significant differences in cell proliferation were observed in any cell line with modified CSMD1 expression when compared to the controls (Figure 2Ei–2Eiii). The same was true after 72 and 96 hours incubation time (not shown). On the other hand, both BT-20 (Figure 3A) and MDA-MB-231 cells expressing CSMD1 (Figure 3B) displayed a significant delay in recovering a scratch wound compared to the control cells after 24 hrs. Accordingly, increased wound recovery was observed in T47D ΔCSMD1 cells (Figure 3C).

The chemotaxis/migration assay measures the ability of cancer cells to move towards an extracellular gradient of serum. BT-20 cells expressing CSMD1 (Figure 3D) and MDA-MB-231 cells expressing CSMD1 (Figure 3E) showed a significant reduction in cellular migration when compared to the control cells. The results also indicated a trend towards an increase in cellular migration for T47D ΔCSMD1 cells (Figure 3F).

**Figure 4:**
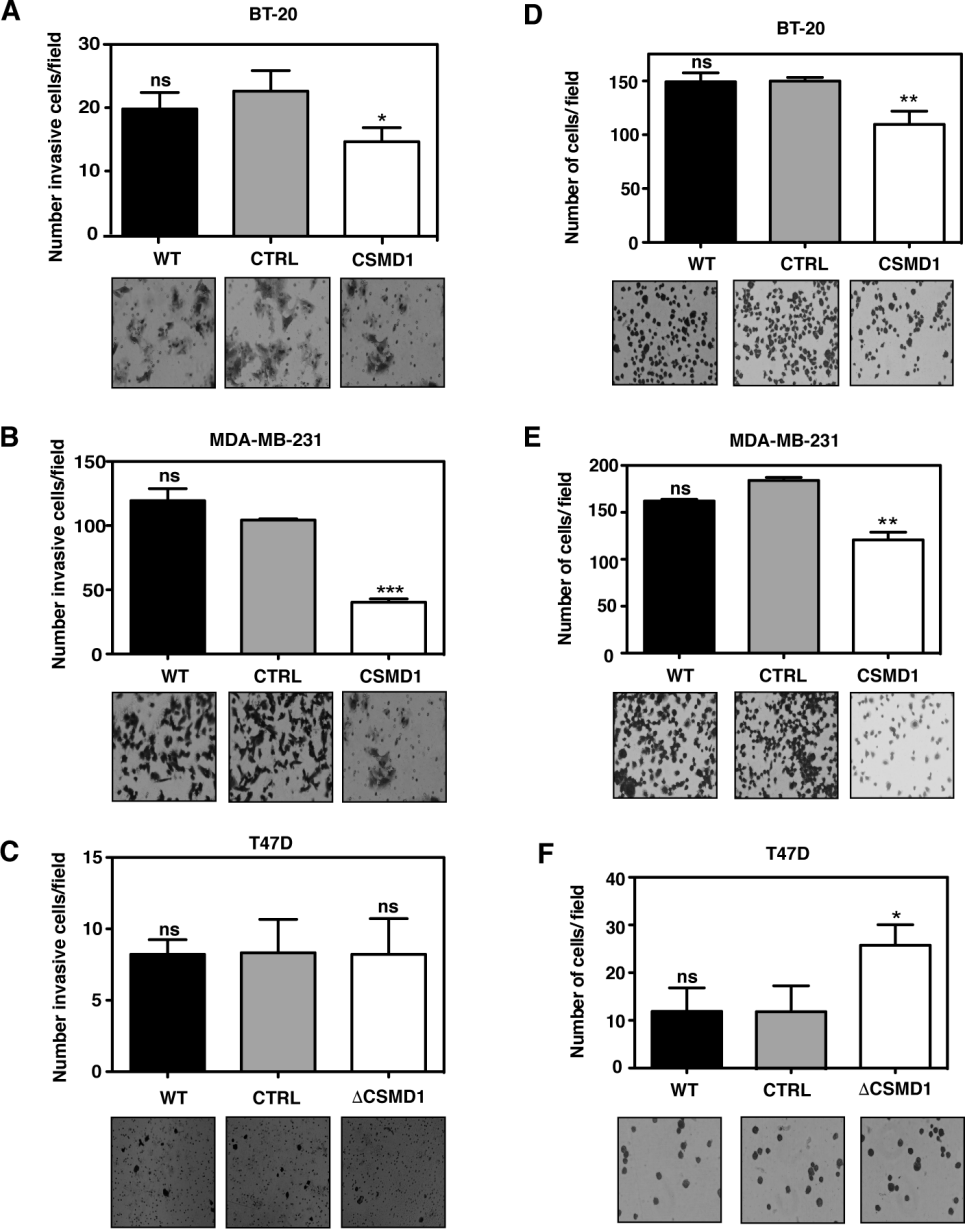
Forced expression of CSMD1 decreases cell invasion and adhesive capacity (**A**–**C**) Cells capable of invading and migrating through a layer of matrigel to the underside of the cell culture insert membranes were photographed and counted after crystal violet staining for BT-20 (A), MDA-MB-231 (B) and T47D cells (C). Data are shown as the mean of cells counts ± SD from 3 independent experiments performed in single inserts. (**D**–**F**) Adherent cells to matrigel were photographed and counted after crystal violet staining for BT-20 (D), MDA-MB-231 (E) and T47D cells (F). Results shown are mean of cells counts ± SD from 3 independent experiments performed in at least four replicate. A one-way ANOVA was used to calculate statistical significance between the CTRL cells and CSMD1 expressing cells; *p < 0.05; **p < 0.01; ***p < 0.001; ****p < 0.0001.

When the invasion assay was performed in BT-20 (Figure 4A) and MDA-MB-231 (Figure 4B) cells, expression of CSMD1 markedly reduced the invasive potential of these cells. However, when the poorly invasive T47D cells were monitored, no significant difference was seen between T47D ΔCSMD1 cells and the controls (Figure 4C).

Taken together, these data indicate that CSMD1 expression in human breast carcinoma cells attenuates their migratory and invasive traits, both of which are hallmarks of tumor cell aggressiveness.

### CSMD1 decreases adhesion

To further explore the effect of altering levels of CSMD1 in transfected cells, the ability to adhere to model extracellular matrix, Matrigel was studied. Expression of CSMD1 significantly inhibited the adhesion of BT-20 cells expressing CSMD1 (Figure 4D) and MDA-MB-231 cells expressing CSMD1 (Figure 4E). The opposite effect was seen for T47D ΔCSMD1 cells, where the adhesive potential was significantly increased (Figure 4F).

### Decreased levels of CSMD1 enhance tumuorigenic potential of cancer cells

**Figure 5:**
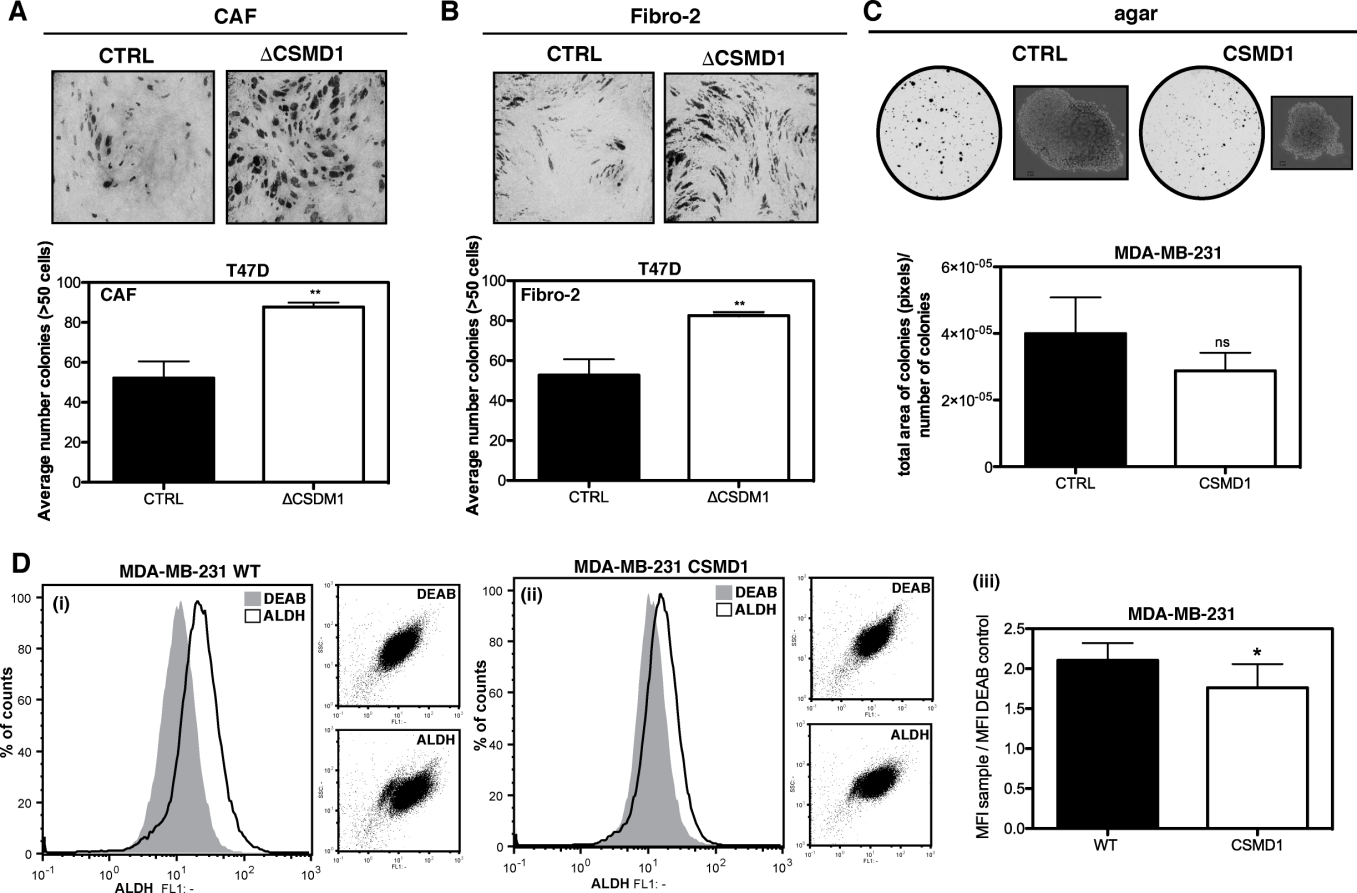
Clonogenicity of ΔCSMD1 cells was stimulated by breast fibroblasts Co-culture of T47D ΔCSMD1 or CTRL cells with either CAFs (**A**) or control fibroblasts (Fibro-2) (**B**) was analysed. Colonies were photographed and counted after toluidine blue staining. Results shown are mean of colony counts ± SD from 3 independent experiments performed in at least three replicate. Unpaired Student's t-test was used to calculate statistical significance between the observed differences; **p < 0.01. Anchorage-independent growth in agar was decreased in CSMD1 expressing cells (**C**) MDA-MB-231 CTRL and CSMD1 expressing cells were cultured in soft agar for 3 weeks. Colonies formed were photographed and counted. Results shown are mean of total area of colonies per number of colonies ± SD analysed in Image J from 3 independent experiments. CSMD1 expressing cells exhibit a smaller sub-population of cells with enhanced ALDH-1 activity (**D**) Flow cytometry analysis of ALDH-1 activity was performed in MDA-MB-231 WT and CSMD1 expressing cells using ALDEFLUOR^®^ assay kit. For all samples in order to determine the ALDH-1 negative cells, fraction of ALDEFLUOR^®^-stained cells was immediately quenched with DEAB, a specific ALDH-1 inhibitor. Left-hand panels show representative histograms of MDA-MB-231 CTRL (i) and MDA-MB-231 CSMD1 (ii) ALDEFLUOR and DEAB treated samples and the corresponding dot plots in the smaller right panels in each case. Right-hand panel (iii) shows mean values for ratio between ALDH-1 and DEAB MFI obtained in three independent experiments. Unpaired Student's *t*-test was used to calculate statistical significance of the observed difference; *p < 0.05.

When co-culturing cancer-associated fibroblasts (CAFs) and T47D ΔCSMD1 cells (Figure 5A), or control fibroblasts and T47D ΔCSMD1 cells (Figure 5B), a significant increase in the number of cell colonies was observed when compared to the T47D CTRL cells. We also found a tendency to form more anchorage-independent colonies in agar for the CTRL cells in comparison with the MDA-MB-231 cells expressing CSMD1 (Figure 5C). To support this observation, we next analyzed a breast cancer stem cell-associated parameter, the activity of ALDH-1 [19, 20]. ALDH-1 negative cells were defined by flow cytometry-based analysis using the ALDH inhibitor DEAB. CSMD1 expression in MDA-MB-231 resulted in a significant relative decrease of ALDH-positive cells by 16% (Figure 5D). Taken together, CSMD1 diminishes the clonogenic properties of cancer cells.

### CSMD1 decreased intracellular signaling potential of cancer cells

**Figure 6:**
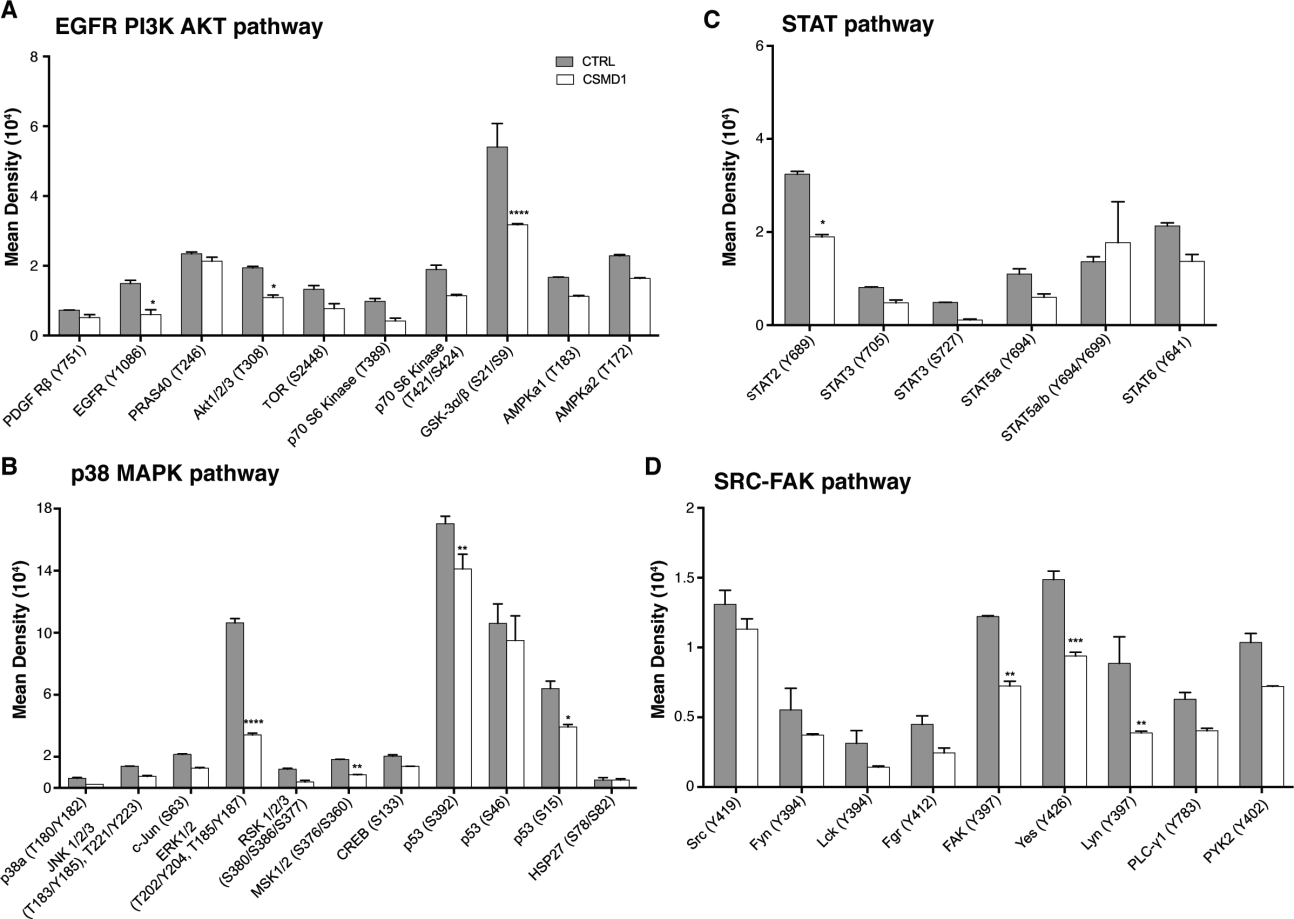
Analysis of phosphorylation of main kinases CTRL and CSMD1 expressing MDA-MB-231 cells were lysed and phosphorylation of panel of kinases was evaluated using Proteome Profiler Array (Phospho-kinase Array Kit). Quantification of mean spot pixel densities obtained for CTRL and CSMD1 cells was depicted in the plots categorized in different pathways (**A**) EGFR PI3K AKT (**B**) p38 MAPK (**C**) STAT (**D**) SRC-FAK pathway. A one-way ANOVA was used to calculate statistical significance between the CTRL cells and CSMD1 cells; *p < 0.05; **p < 0.01; ***p < 0.001.

To investigate which phosphorylation events that are altered by expression of CSMD1, we utilized a phospho-kinase antibody array encompassing 43 kinase phosphorylation sites. Cells were serum starved for 30 min and then lysed to examine protein phosphorylation. Following quantification of the densities of individual dots corresponding to a panel of phosphorylated kinases, we observed an overall diminished signaling potential in the MDA-MB-231 breast cancer cells expressing CSMD1 in comparison with CTRL cells. Kinases, which were phosphorylated to a lower degree in the presence of CSMD1 in statistically significant manner included: epidermal growth factor receptor (EGFR), Akt1/2/3, glycogen synthase kinase-3 (GSK-3), extracellular signal–regulated kinases (ERK1/2), mitogen- and stress-activated kinases-1 and -2 (MSK 1/2), oncogene p53, focal adhesion kinase (FAK), SRC kinases Lyn and Yes, as well as STAT2 (Figure 6A–6D). We grouped the kinases according to their roles in different major signaling pathways that play prominent roles in breast cancer progression [21–24]. We found that the EGFR/PI3K/AKT, p38 MAPK and SRC-FAK pathways were the most affected pathways, whereas the effect on the STAT pathway was moderate.

### CSMD1 expressing cancer cells exhibited low incidence of metastatic *in vivo*

**Figure 7:**
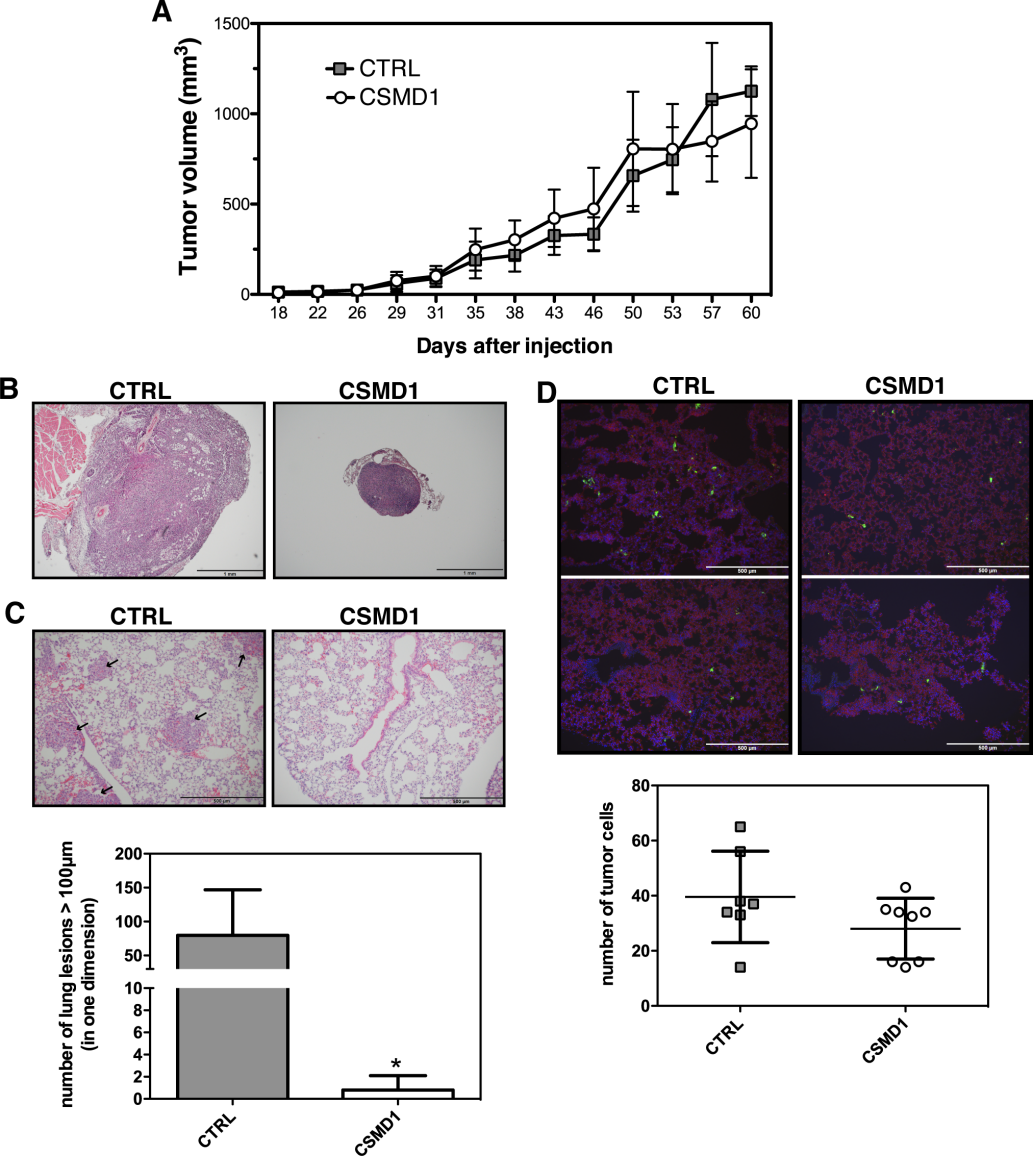
CSMD1 expressing MDA-MB-231 cells do not form metastatic foci in xenograft model (**A**) The CTRL and CSMD1-transfected cells were injected into the mammary fat pad of SCID (CB-17/Icr-*Prkdc^scid^*/Rj) mice and tumor volumes were measured for 60 days or until the tumors reached 1 cm^3^. The data represents mean tumor volumes ±SD of the 10 animals in each group (**B**, **C**) The lymph nodes and lungs were extracted and stained for hematoxylin and eosin (B) Representative hematoxylin and eosin staining of lymph nodes indicating the difference in size between the two groups. (C) Representative sections of lung tissue with black arrows indicate the metastatic lesions. Results shown are means of metastatic lesions bigger than 50 μm counted on each slide ± SD (*n* = 5 per group). Unpaired Student's *t*-test was used to calculate statistical significance of the observed difference; *p < 0.05 (**D**) Immunostaining of lung tissue for CD31 (red) and extravasated tumor cells (green) after intravenous injection of fluorescent labelled CTRL and CSMD1 expressing MDA-MB-231 cells. Results shown are means of number of metastatic cells counted on each slide ± SD (*n* = 8 per group).

To examine the role of CSMD1 *in vivo*, control- or CSMD1-transfected MDA-MB-231 cells were injected into the mammary fat pad of SCID mice. No significant differences in the tumor growth of the primary tumors were observed between the two groups of mice (Figure 7A). However, in the control group we observed 3–4 fold enlarged lymph nodes in 5 of 9 animals suggesting lymph node metastases. In contrast, in the CSMD1 expressing group none of 9 animals had enlarged lymph nodes (Figure 7B). We also observed a dramatic decrease in the number of metastases formed in lungs when comparing CSMD1 expressing tumors and controls (Figure 7C).

We then evaluated the ability of cancer cells to extravasate to the site of metastasis. For this purpose fluorescently labeled control- and CSMD1-transfected MDA-MB-231 cells were injected intravenously into SCID mice and counted in lung tissue sections taken after 24 h. The data suggest that the expression of CSMD1 does not influence the extravasation process, as the number of fluorescent cancer cells that entered via circulation to the lungs was not significantly different between the two groups (Figure 7D). Even though there was a small trend towards diminished extravasation of cancer cells to the lung in the CSMD1 group, this cannot explain the drastic difference in the occurrence of metastatic foci observed in the previous experiment. These observations together lead to the conclusion that CSMD1 likely influences the rate of escape of tumor cells from the primary tumor.

## DISCUSSION

CSMD1 is a large transmembrane protein suggested to act as tumor suppressor on the basis of genetic findings, i.e. mutations and deletions in the CSMD1 gene are associated with different types of cancer including breast cancer [7, 25]. These deletions are generally associated with poor prognosis and a more malignant cancer phenotype. In this study, we provide direct experimental evidence that CSMD1 acts as a tumor suppressor gene, and we revealed some mechanisms responsible for this function.

Consistent with the previously observed presence of CSMD1 mRNA in whole breast tissue [3], the current *in situ* hybridisation analysis of CSMD1 mRNA revealed the expression of CSMD1 in ductal epithelial cells. The tumor suppressor role of CSMD1 was strongly supported by our clinical observation that breast cancer tissues had low levels of CSMD1 compared with normal mammary tissues, and the fact that low levels of the transcript were significantly associated with a shorter survival of the patients. This was also supported by data from an independent cohort (KM plot). A similar observation was previously made using immunohistochemistry in which low CSMD1 protein levels were linked to high tumor grade and shorter overall survival [7].

This current report is the first to study the effect of CSMD1 expression on the biological properties of epithelial breast cancer cells. The panel of breast cancer cells used has served as a suitable model to demonstrate that CSMD1 acts as a tumor suppressor. First we found that changes in the expression of CSMD1 did not modify the growth of the cancer cells, which simplified interpretation of the following migration and invasion assays. Metastasis is a complex process involving a number of steps that must be accomplished for the cancer cell to effectively penetrate the basement membranes. The atypical motile behavior that cancer cells display is a common feature among all types of solid tumors. We therefore performed wound healing assays to study the random movement of cancer cells on a solid surface. The closure of the wound occurred notably faster in cells lacking CSMD1 than in the expressing cells. A similar effect was observed when investigating directional migration in response to a serum gradient, indicating that CSMD1 inhibits epithelial cell motility. Since cell migration requires an active interaction between the cells and the substrate on which they are attached, we next examined adhesion of the cells with altered levels of CSMD1. Unexpectedly, expression of CSMD1 decreased cellular adhesion to an artificial basement membrane. The opposite was observed when silencing CSMD1. Apparently, the observed effect on attachment was minor compared to the opposite effect on cell migration and therefore not observed in the latter assays. The decreased adhesion in the presence of CSMD1 could be beneficial in the later step of metastasis when cancer cells attach to the target tissue. Invasion refers to the ability of the cancer cells to penetrate the surrounding tissues through the degradation of the basement membrane and the extracellular matrix. CSMD1 reduced the invasive potential of CSMD1 expressing cells, particularly in the highly invasive MDA-MB-21 cells. For the weakly invasive BT-20 cells, the effect was less pronounced. Considering that T47D cells are a non-invasive cell line, the lack of effect upon deletion of CSMD1 was expected.

Over the last decade, it has become apparent that the tumor microenvironment is a key modulator of tumor progression and metastasis. CAFs are the most abundant cell type in the tumor stroma [26]. They are associated with numerous steps of the metastatic cascade, both in the primary site and in the secondary tumor in a distant organ [27, 28]. Thus, we studied the effect of CAFs and control fibroblasts on tumor cells with modified levels of CSMD1 on the survival and proliferation using direct physical contact between cancer cells and fibroblasts. BT-20 and MDA-MB-231 cells did not form visible colonies but rather grew in layers on top of fibroblasts. In contrast, T47D cells lacking CSMD1 were significantly affected by the presence of both types of fibroblasts and showed enhanced cell growth and clonogenicity compared to control T47D cells expressing CSMD1.

It is now commonly accepted that a subset of cells within a tumor possess unique tumorigenic properties similar to stem cells, also responsible for the metastasis in a secondary site [29]. Interestingly, the ability of cells to initiate spheroid-colony growth in soft agar was inhibited in CSMD1 expressing cells, suggesting altered stem-like characteristics of cancer cells. Furthermore, CSMD1 expression resulted in decreased ALDH-1 activity, a key enzyme involved in stem cell differentiation [19], likely translating to lower tumorigenic capacity.

CSMD1 has a small cytoplasmic tail of 56 amino acids with a putative tyrosine phosphorylation site. Therefore, CSMD1 may serve as a receptor or a co-receptor for unknown ligands and may be involved in signal transduction. Our data offers support for the involvement of CSMD1 in different signaling pathways with an overall decrease of the cell signaling potential, correlating with the observed functional properties of cancer cells expressing CSMD1.

Observations from the patient tissues and the *in vitro* data obtained with three different cell lines were confirmed by *in vivo* experiments with CSMD1 expressing breast cancer cells. Here, we found no measurable effect of CSMD1 expression on the growth of primary tumors but we detected a striking difference in the ability of the cancer cells to metastasize to secondary sites. Thus, MDA-MB-231 cells expressing CSMD1 essentially lost the ability to metastasise, while control cells formed readily metastases in lymph nodes and lungs. We did not observe differences in lymphocyte infiltration in tumors sections (staining for CD45; data not shown), indicating that the tumor-suppressing activities of CSMD1 are not linked to its complement inhibitory function. Furthermore, experiments showing no difference in extravasation of CSMD1 expressing cells into lungs compared to control cells, indicated that the difference lies in the ability of the cells to detach from the primary tumor. This is consistent with our observations that CSMD1 expressing cells are less motile and invasive, displaying less stem cell-like properties.

In summary, we provide experimental evidence for the role of CSMD1 as a tumor suppressor *in vitro* and *in vivo* in the progression of breast cancer. We revealed that expression of CSMD1 significantly reduced cell motility and migration, adhesion and invasion, as well as the tumorigenic and signaling potential of human breast cancer cells. Conversely, knockdown of CSMD1 enhanced cell motility and cell adhesion as well as clonogenic potential. Furthermore, expression of CSMD1 *in vivo* resulted in diminished formation of metastatic foci in a xenograft model likely due to impaired rate of escape of tumor cells from the primary tumor. Collectively, our results confirm that low levels of CSMD1 in breast cancer cells are associated with more aggressive cancer cell behavior, hence demonstrating the role of CSMD1 as tumor suppressor gene.

## MATERIALS AND METHODS

### Cell lines, culture conditions and mammary tissues

The human breast cancer cell lines BT-20, MDA-MB-231 and T47D were purchased directly from ATCC and all the experiments were performed on cultures within no more than 5 passages. Stable transfectants were cultured with addition of 3 μg/ml puromycin (BT-20 and MDA-MB-231; Invitrogen) or 5 μg/ml of blasticidin S (T47D; Invitrogen). Generation of human breast carcinoma-associated fibroblasts (CAFs) and the corresponding control fibroblasts was described [30]. All cells were maintained in DMEM (Thermo Scientific) supplemented with 10% fetal bovine serum (FBS) and penicillin/streptomycin. All cells were monthly tested for *Mycoplasma* contamination with VenorGEM Classic kit (Minerva Biolabs).

Fresh frozen mammary tissues (normal, *n* = 32 and tumor, *n* = 127) from patients with breast cancer were collected immediately after surgery, under the research ethics approval from the Southeast Wales Research Ethics Committee, and stored at −80^°^C until used. Patients were followed up routinely in the clinics with a median follow-up at 120 months and their clinical characterisation was published previously [31].

### *In situ* hybridisation staining

*In situ* hybridization was used for detection of CSMD1 mRNA in paraffin-embedded normal breast tissue sections and pellets of BT-20 CSMD1 or control-transfected cells. Tumor material was obtained from female patients diagnosed with invasive breast cancer at Malmö University Hospital [32]. Ethical permission was obtained from the Lund University Regional Ethics Board, ref. no. 445/2007 whereby written consent was not required and patients were offered the possibility to opt out. After baking the slides at 60°C for 1 hour and deparaffinisation in xylene followed by dehydration in absolute ethanol, CSMD1-specific RNA, peptidylprolyl isomerase B, PPIB (cyclophilin) housekeeping gene RNA (positive control) and dihydrodipicolinate reductase from *Bacillus subtilis*, DapB RNA (negative control) were detected using RNAscope 2.0 BROWN assay (Advanced Cell Diagnostics) [33]. Pictures were taken using an Aperio ScanScope (Leica Microsystems). The images were processed in Adobe Photoshop in order to distinguish condensed nucleoli (grey/violet) from the signal from the RNA probes (brown) [34].

### Modification of CSMD1 expression in breast cancer cells

For over-expression, a p509 plasmid containing CSMD1 cDNA (DNA 2.0) was transfected into the cells, in parallel with an empty p509-GFP plasmid. The protein sequence was exactly as in GenBank: AAQ88541.1, but the DNA sequence was optimized for high expression. Cells were transfected using Lipofectamine 2000 (Invitrogen) or FuGENE HD (Promega) and clones picked after selection in antibiotics. The cells expressing CSMD1 were designated as CSMD1, those containing the control p509-GFP plasmid were designated as CTRL, unaltered wild type cells were designated as WT. T47D cell line demonstrating reduced CSMD1 levels was generated previously using a ribozyme transgene system [3] and designated as ΔCSMD1.

### RNA extraction, PCR and real time qPCR

RNA was isolated from picked colonies using Trizol (Invitrogen). The quality of the RNA was assessed using an Experion RNA StdSens Analysis Kit (Bio-Rad). RNA was converted to cDNA using 2.5 μM Oligo(dT) primer, 24 U RnaseOUT riblonuclease inhibitor, 200 U Superscript III reverse transcriptase (Invitrogen) and 0.5 mM dNTPs (Fermentas).

Conventional PCR was performed in a S1000 Thermal-Cycler (Bio-Rad) using REDTaq^®^ ReadyMixTM PCR Reaction mix (Sigma-Aldrich). Primer sequences for CSMD1 were: forward primer 5′- AGATGCTGCCGTCAAAAGATGGAT-3′ and reverse 5′- TCACTTTGTCTGGGTCCGTTGTTG-3′. GAPDH was used as internal loading control. To quantify transcript copy number of CSMD1, Taqman assays (Applied Biosystems) were used. Samples were tested in triplicate and CSMD1 (Hs00899098_m1) gene expression level was calculated using the ΔCT method after normalisation with the geometric mean of the three housekeeping genes; cyclophilin A (Hs99999904_m1), TATA box binding protein (Hs0042761_m1) and hypoxanthine phosphoribosyltransferase 1 (Hs99999909_m1).

For mammary samples, tissues were homogenised and total RNA was extracted, quantified and subjected to a reverse transcription kit (PrimerDesign). Quantification of the CSMD1 and the house keeping actin transcripts were carried using the Ampliflor technology in which a uniprimer with a Z-sequence (underlined in the following) was used as a probe. The primer sequence for CSMD1 and actin were respectively (5′ - 3′): aggagatgagaggccaag vs actgaacctgaccgtacaccccataaactgtcaacg, and ctgagtacgt cgtggagtc vs actgaacctgaccgtacacagagatgaccctttg

### Analysis of cell surface CSMD1 by flow cytometry

Cells were detached with versene and incubated with rabbit anti-CSMD1 antibody generated against recombinantly expressed CCP2/CUB3 domains (Agrisera). The cells were then washed twice and incubated with FITC-labelled swine anti-rabbit IgG diluted (Dako) for 30 min at room temperature, and analysed by flow cytometry (Partec Cyflow) and FlowJo software.

### Cell viability assay

Cells were seeded in a 96-well plate and grown under standard conditions for 24 hours and 100 μl of fresh medium with 10% of alamarBlue (Invitrogen) was added to the cells for 6 hours. Absorbance was determined at a wavelength of 570/600 nm using a microtiter plate reading spectrophotometer (Cary 50 Bio).

### Wound healing assay

Cells were grown to confluency in a 6-well plate and the monolayer was scratched with a sterile pipette tip to create a wound. Cells were photographed immediately after wounding and every 2/4/6/24 hours with EVOS FL inverted microscope at ×10 magnification. Image J was used to determine the wound area and percentage wound closure was calculated.

### Chemotaxis/migration assay

The motility of cancer cells was studied using cell culture inserts (8 μm or 3 μm pore size) placed into a 24-well plate (Corning). Cells were seeded in the inserts with 1–10% FBS chemotactic gradient. After incubation period, the cells that had migrated through the porous membrane were fixed in 4% formaldehyde for 10 minutes before being stained in 0.5% crystal violet (Merck Millipore). The cells were then photographed at ×40 magnification. Three random fields were counted for each duplicate insert.

### Matrigel adhesion assay

The cell-matrix attachment was carried out as previously described [35]. Briefly, cells were added to 96-well plate coated with Matrigel (BD Bioscience) and incubated at 37°C for 40 minutes. The membrane was washed 5 times, then fixed in 4% formaldehyde for 10 minutes before being stained in 0.5% crystal violet. The number of adherent cells was counted from 2 random fields per well and minimum of 4 replicate wells per sample at ×40 magnification.

### Invasion assay

Invasiveness was determined using 24-well plate BioCoat Matrigel chambers (Corning). Cells were seeded in the inserts with 1–10% FBS chemotactic gradient. The plates were then incubated for 24/48/72 hours at 37^°^C and the matrigel layer together with the non-invasive cells was removed with a cotton swab. The cells that have migrated through the Matrigel and porous membrane of the insert were fixed in 4% formaldehyde for 10 minutes before being stained in 0.5% crystal violet. The cells were then visualised at ×40 magnification. Three random fields were counted for each test sample.

### Clonogenic assay

CAFs and control fibroblasts were seeded in a 96-well plate (5000 cells per well) in order to create a confluent monolayer of fibroblast. Then, 50 cells were seeded on top of the fibroblasts. After 10 days the cells were fixed with 70% ethanol for 10 minutes before being stained in 0.1% toluidine blue. The colonies were photographed at ×4 magnification. A colony was defined as a cluster of minimum 50 cancer cells.

### Soft agar colony formation

A 0.6% (w/v) bottom layer of low melting point agarose in normal medium was prepared in Petri dishes (90 mm). On top, a layer of 0.3% agarose containing 30 000 cells was placed. The cells were fed twice a week with normal medium. After 4 weeks, colonies were stained with 3-(4,5-dimethylthiazol-2-yl)-2,5-diphenyl tetrazolium bromide, photographed using CCD camera and counted with Image J.

### Identification of aldehyde dehydrogenase (ALDH-1) positive cells

The Aldefluor assay was carried out according to guidelines (StemCell Technologies). Briefly, 1*10^6^ cells were stained with Aldefluor reagent (1 μM) in assay buffer. As a parallel negative control the reaction was stopped with 50 mM of the specific ALDH-1 inhibitor diethylaminobenzaldehyde (DEAB). Cells were incubated for 1 hour at 37°C, pelleted at 400 g for 5 min, resuspended in 1 ml assay buffer and stored on ice prior to analysis using flow cytometry.

### Proteome profiler assay

Activity of signaling pathways was investigated using the Proteome Profiler arrays (Phospho-kinase Array Kit; R&D Systems). These are nitrocellulose membranes with antibodies against 43 kinase phosphorylation sites and two control antibodies spotted in duplicates. Cell lysates from 30 min serum starved CTRL and CSMD1 expressing cells were prepared in the provided lysis buffer. In total, 300 μg of protein was used for each array and incubated with the membranes overnight at 4°C. The detection was conducted according to manufactures' instructions. Densities of individual dots corresponding to a phosphorylated kinase were measured by Image J software to compare CTRL and CSMD1 expressing cells.

### Animal experiments

### Orthotopically injection into the mammary fat pad

MDA-MB-231 CTRL or CSMD1 cells were orthotopically transplanted (5 × 10^6^ cells/animal) into the 4th right inguinal mammary fat pad of 8 weeks old SCID (CB-17/Icr-*Prkdc*^scid^/Rj) mice. Each group consisted of 10 animals. Tumor growth was followed twice per week by measuring each tumor in two dimensions using a caliper. The tumor volume was calculated using the formula V = dim1*dim2^2^*π/6; dim1 > dim2. Mice were sacrificed when tumors reached the size of 1 cm^3^. Mice were injected with an overdose of tribromoethanol (Sigma) and perfused with PBS. Tumors, lungs and lymph nodes were saved for further analyses.

### Intravenous injection of cancer cells

To analyze tumor cell extravasation 5 × 10^5^ fluorescently labeled (CellTracker™ Green CMFDA, Molecular Probes) MDA-MB-231 CTRL and CSMD1 cells were injected in the tail vein and allowed to circulate for 24 hours. Mice were perfused and sacrificed. Cryopreserved lungs were sectioned and analyzed for extravasated labeled cells (20× magnification).

### Histology and staining

Mice were heart perfused with PBS. Organs were fixed in 4% paraformaldehyde, dehydrated and paraffinized prior to use by subsequent incubation in xylene, ethanol and water. For cryopreservation organs were kept in 30% sucrose at 4°C over night, followed by embedding in OCT cryomount (Histolab). Tissues for RNA extraction were snap-frozen in liquid nitrogen. For metastases evaluation we used paraffin embedded right lungs of 5 mice per group. The metastatic burden was assessed by serial sectioning of the entire lung following hematoxylin and eosin (Histolab) staining on every 30th section. Metastatic lesions larger than 50 μm were counted on each slide. Sections for immunostaining were fixed in ice-cold aceton followed by washing with PBS. After blocking with serum free protein block (DAKO) 90 min at RT, antibody against CD31 (Clone Mec 13.3, dilution 1:200, BD pharmingen) was applied in PBS supplemented with 1 % BSA at 4°C over night. After washing Alexa flour 594 donkey anti-rat IgG (Life Technologies, 1:1000) was applied for 2 hours at RT. Slides were mounted in DAPI containing mounting medium. Imaging was done using an Olympus BX63 microscope with Olympus DP80 camera.

### RNA extraction

Snap frozen material was disrupted using TissueLyser LT (Qiagen), 5 mm stainless steel beads (Qiagen) and reagent DX (Qiagen). The lysate was further homogenized using QIAshredder (Qiagen) and RNA was extracted using RNeasy mini kit (Qiagen) with on column DNAse digest (Qiagen) and additional washing steps. The quality of the RNA was assessed using an Experion RNA StdSens Analysis Kit (Bio-Rad).

### Statistical analyses

Statistical analyses were performed using GraphPad Prism 5.0 and SPSS with methods listed in Figure legends.
