# Correction: Complement inhibitor CSMD1 acts as tumor suppressor in human breast cancer

**DOI:** 10.18632/oncotarget.28426

**Published:** 2023-05-19

**Authors:** Astrid Escudero-Esparza, Michael Bartoschek, Chrysostomi Gialeli, Marcin Okroj, Sioned Owen, Karin Jirström, Akira Orimo, Wen G. Jiang, Kristian Pietras, Anna M. Blom

**Affiliations:** ^1^Department of Translational Medicine, Lund University, Malmö, Sweden; ^2^Department of Laboratory Medicine, Lund University, Lund, Sweden; ^3^Department of Medical Biotechnology, Medical University of Gdańsk, Gdańsk, Poland; ^4^Cardiff China Medical Research Collaborative, Cardiff University School of Medicine, Cardiff University, Cardiff, UK; ^5^Department of Clinical Sciences, Lund University, Lund, Sweden; ^6^Department of Pathology and Oncology, Juntendo University School of Medicine, Tokyo, Japan; ^*^These authors contributed equally to this work

**This article has been corrected:** In Figure 4B, the image of MDA-MB-231 cells expressing CSMD1 is an accidental duplicate of the image showing invaded BT-20 cells expressing CSMD1 in Figure 4A. The correct Figure 4, produced using the original data, is shown below. The authors declare that these corrections do not change the results or conclusions of this paper.

Original article: Oncotarget. 2016; 7:76920–76933. 76920-76933. https://doi.org/10.18632/oncotarget.12729

**Figure 4:**
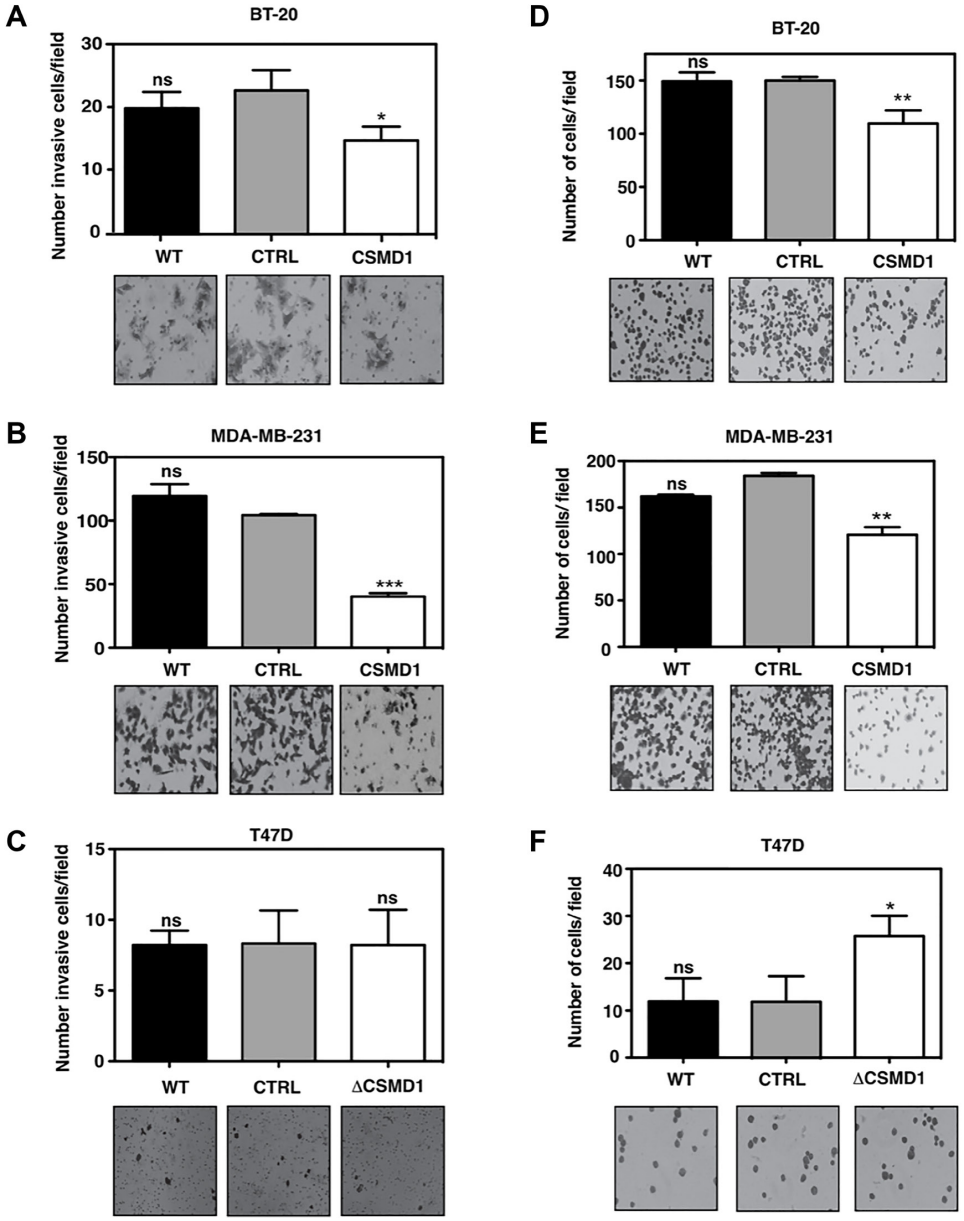
Forced expression of CSMD1 decreases cell invasion and adhesive capacity. (**A**–**C**) Cells capable of invading and migrating through a layer of matrigel to the underside of the cell culture insert membranes were photographed and counted after crystal violet staining for BT-20 (A), MDA-MB-231 (B) and T47D cells (C). Data are shown as the mean of cells counts ± SD from 3 independent experiments performed in single inserts. (**D**–**F**) Adherent cells to matrigel were photographed and counted after crystal violet staining for BT-20 (D), MDA-MB-231 (E) and T47D cells (F). Results shown are mean of cells counts ± SD from 3 independent experiments performed in at least four replicate. A one-way ANOVA was used to calculate statistical significance between the CTRL cells and CSMD1 expressing cells; ^*^ *p* < 0.05; ^**^ *p* < 0.01; ^***^ *p* < 0.001; ^****^ *p* < 0.0001.

